# A Pilot Study of Short-Duration Sputum Pretreatment Procedures for Optimizing Smear Microscopy for Tuberculosis

**DOI:** 10.1371/journal.pone.0005626

**Published:** 2009-05-20

**Authors:** Peter Daley, Joy Sarojini Michael, Kalaiselvan S, Asha Latha, Dilip Mathai, K. R. John, Madhukar Pai

**Affiliations:** 1 Department of Medicine, Christian Medical College Vellore, Vellore, India; 2 Department of Microbiology, Christian Medical College Vellore, Vellore, India; 3 Department of Community Health, Christian Medical College Vellore, Vellore, India; 4 Department of Epidemiology, Biostatistics and Occupational Health, McGill University, Montreal, Quebec, Canada; National Institute for Infectious Diseases (INMI) L. Spallanzani, Italy

## Abstract

**Background:**

Direct sputum smear microscopy for tuberculosis (TB) lacks sensitivity for the detection of acid fast bacilli. Sputum pretreatment procedures may enhance sensitivity. We did a pilot study to compare the diagnostic accuracy and incremental yield of two short-duration (<1 hour) sputum pretreatment procedures to optimize direct smears among patients with suspected TB at a referral hospital in India.

**Methodology/Findings:**

Blinded laboratory comparison of bleach and universal sediment processing (USP) pretreated centrifuged auramine smears to direct Ziehl-Neelsen (ZN) and direct auramine smears and to solid (Loweinstein-Jensen (LJ)) and liquid (BACTEC 460) culture. 178 pulmonary and extrapulmonary TB suspects were prospectively recruited during a one year period. Thirty six (20.2%) were positive by either solid or liquid culture. Direct ZN smear detected 22 of 36 cases and direct auramine smears detected 26 of 36 cases. Bleach and USP centrifugation detected 24 cases each, providing no incremental yield beyond direct smears. When compared to combined culture, pretreated smears were not more sensitive than direct smears (66.6% vs 61.1 (ZN) or 72.2 (auramine)), and were not more specific (92.3% vs 93.0 (ZN) or 97.2 (auramine).

**Conclusions/Significance:**

Short duration sputum pretreatment with bleach and USP centrifugation did not increase yield as compared to direct sputum smears. Further work is needed to confirm this in a larger study and also determine if longer duration pre-treatment might be effective in optimizing smear microscopy for TB.

## Introduction

Direct Ziehl-Neelsen (ZN) sputum smear microscopy for the detection of acid fast bacilli (AFB) remains the most important diagnostic test for tuberculosis (TB) in high burden countries. Although rapid, specific, and appropriate for laboratories with minimal infrastructure, sputum microscopy failed to detect 56% of the estimated global burden of new TB cases in 2006.[1] The proportion of missed cases may be higher among HIV infected patients, who are often smear negative despite multiple smears.[2] The analytical sensitivity of direct sputum smear microscopy is approximately 10,000 bacilli per millilitre of sputum, much less than culture methods.[3] The Global Plan to Stop TB recognizes the limitations of sputum smear microscopy and mandates further research into the optimization of this technique.[4], [5]

One approach to the improvement of sputum smear microscopy is the application of chemical or physical pretreatment (“sputum processing”) procedures to disrupt sputum structure, separate clumps of mycobacteria, and concentrate bacilli, thereby increasing the probability of their detection. Some pretreatment procedures may permit subsequent culture, but some kill all mycobacteria. There is some evidence that a single pretreated smear may detect as many cases as three direct smears.[6]

Several approaches to sputum pretreatment have been summarized in a recent meta-analysis.[7] Twenty-two of 59 studies in this meta-analysis reported sensitivity of smears as compared to culture, and the remaining studies reported incremental yield as compared to direct smear. Centrifugation with various prior chemical pretreatments (32 studies) increased sensitivity by 18% (range −3 to +39%). Centrifugation with bleach (17 studies) increased sensitivity by 15% (range +1 to +38%). Although sputum pre-treatment appeared to be effective, this meta-analysis could not separate the effect of chemicals (e,g, bleach) from the various physical methods (e,g, centrifugation) used. A narrative review of the bleach method stated that there is enough evidence to support the evaluation and introduction of bleach microscopy in settings where mycobacterial culture is not widely used.[8]

However, experts have highlighted the lack of available evidence and the operational challenges associated with the application of chemical and physical pretreatment methods in resource-limited settings, including biosafety concerns, need for equipment such as centrifuges, and lack of standardization and quality assurance of methods.[9], [10] These reports called for further research into the performance characteristics of various smear optimization methods under carefully controlled conditions before global policy supporting pretreatment can be adopted.

It is important that sputum pretreatment procedures do not cause prolonged delay in the reporting of results, or they will not be operationally feasible for national TB programs in high burden countries. For example, although pretreatments requiring overnight incubation may be effective [11], they cannot provide results to the patient on the same day that sputum is submitted. Therefore, we feel it is important to perform field evaluation of short-duration pretreatment methods that provide a turn around time for sputum processing of less than one hour.

The objective of our pilot study was to evaluate the performance of various new diagnostic tests for TB in India, to identify candidate assays that may be suitable for further development and study in larger clinical trials. This study evaluated the improvement in sensitivity, as well as incremental yield, of two combinations of short duration chemical and physical pretreatment procedures for smear microscopy in a large referral hospital in south India. We compared bleach and universal sediment processing (USP) as chemical methods, both used in combination with centrifugation

Bleach is one of the most widely studied chemical methods.[7] USP has been described from India, but is yet to be replicated in other Indian mycobacteriology laboratories.[12], [13]

We evaluated the diagnostic performance of pretreated and direct smears as compared to solid and liquid culture and analysed incremental yield of pretreated smears as compared to direct ZN and direct auramine smears. By designing our trial according to the STARD guidelines [14], including a rigorous blinding procedure, we aimed to avoid methodological problems associated with previous reports.[15]

## Methods

### Ethics

The study protocol was approved by the institutional review boards at both CMC Vellore and McGill University, Canada. All patients provided written informed consent for participation.

### Setting

Our study was conducted at the Christian Medical College, a 2200 bed tertiary referral center in Vellore, a town in Tamil Nadu state in South India. Vellore district has an annual TB case detection rate of 144/100,000[10]. The Microbiology department at CMC, Vellore, examines an average of 120 smears per day by fluorescent staining, and is externally accredited by the Central TB Division in Delhi and the supranational reference laboratory at the TB Research Center in Chennai.

### Participants

Patients with suspected pulmonary or extrapulmonary TB were prospectively recruited by a trained research officer, from both TB clinic and medical wards. Patients greater than 18 years old were included if the treating physician felt the diagnosis of TB was possible, and the patient was willing to comply with the study protocol including an HIV test and follow-up visit. Symptom criteria were outlined clearly. Pulmonary TB suspects had two or more of: cough for two weeks, weight loss of 10% of healthy body weight, or symptoms of fever for two weeks (or one measured temperature above 38.5°C). Extrapulmonary TB suspects had one focal symptom lasting three weeks and weight loss of 10% of healthy body weight and/or symptoms of fever for two weeks (or one measured temperature above 38.5°C). Patients were excluded if they had received any TB treatment in the last six months, or if any specimen was submitted in formalin, since culture would not be possible.

Two specimens were collected from each patient. The first specimen was collected at recruitment, treated with NALC/NaOH and used for culture tests. The sputum smear optimization procedure was performed on a single morning sputum sample of at least 4 ml in volume, collected at the second visit of the patient, 48 hours after recruitment, but before the initiation of treatment.

### Laboratory Methods

The specimen was carefully homogenized with a Pasteur pipette for one minute, followed by vortexing for 30 sec–1 minute, to ensure uniform distribution of bacilli. After homogenization, the specimen was divided into three parts. The first 0.5 ml was used for direct ZN and auramine smears, 2 ml was used for pretreatment experiments, and the residual was stored at −70°C.

### Direct Smears

Direct smears were prepared with new microscope slides and fixed using 5% phenol in 70% alcohol for 5 minutes. Auramine smears were prepared with auramine stain for 15 minutes, then rinsed briefly with water, flooded with acid alcohol for 30 seconds then rinsed with water and counterstained with potassium permanganate (Potassium Permanganate 1.0 gm, distilled water 1000 ml) for 1 minute. Slides were examined by a single technologist using a 20× objective on a Leica DMLS microscope , viewing at least 30 fields, with positive and negative control slides examined twice per week.[16]

Ziehl-Neelsen smears were prepared with 0.32% carbol fuchsin, heated gently for 5 minutes until steam rose, washed with tap water and flooded with 3% HCl acid-alcohol for 1 minute, washed and flooded with 1% methylene blue for 1 minute. Slides were examined by a single technologist using a 100× oil immersion objective, viewing at least 100 fields, with positive and negative control slides examined twice per week.[16]

Smears for acid fast bacilli were interpreted using standard criteria proposed by the International Union Against TB and Lung Disease and shown in Table 1.[17]

**Table 1:** Interpretation and reporting of microscopy results [17]

	Number of Acid Fast Bacilli Seen by Staining Method	
Report	Ziehl-Neelsen Stain	Auramine Stain
	1000×	200×
Negative	Zero in 100 HPF	Zero in 30 HPF
Scanty	1–9 AFB in 100 HPF (report exact number)	1–29 AFB in 30 HPF
1+	10–99 AFB in 100 HPF	30–299 AFB in 30 HPF
2+	1–10 AFB per HPF (on average)	10–100 AFB per HPF (on average)
3+	>10 AFB per HPF (on average)	>100 AFB per HPF (on average)

AFB = Acid Fast Bacilli

HPF = High Powered Field

### Pretreatment

Each 2 ml specimen was divided into four equal parts using a Pasteur pipette. Aliquots in 1.8 ml cryovials were labelled with a five digit random number by the laboratory investigator. One aliquot was used for bleach pretreatment and one for USP pretreatment.

Bleach pretreatment was performed using commercial 5% NaOCl (Qualigens, Glaxo, Mumbai). An equal amount of bleach was added to 0.5 ml of sputum in a 50 ml Falcon tube, which were vortexed well and incubated at room temperature for 15 minutes, then distilled water was added to a final volume of 50 ml, and inverted 3–4 times.

USP pretreatment was performed using USP solution as described.[18] USP is a combination of a chaotropic agent (guanidinium hydrochloride) with a mucolytic agent and detergents. The original method involved a variable length of incubation in USP followed by centrifugation. The sediment was washed a second time with USP if it did not reduce to 10% of the volume of the original specimen.

Two volumes of USP solution were added to 0.5 ml sputum in a 50 ml Falcon tube, which was vortexed well and incubated at room temperature for 15 minutes, then distilled water was added to a final volume of 50 ml.

Centrifugation was performed for one bleach and one USP aliquot, at 3000g for 15 minutes. The supernatant was decanted by pouring with a Pasteur pipette, and smears prepared from the mixed sediment.

### Liquid Culture

Liquid culture was performed using BACTEC 460 MB (Becton-Dickenson, Hryana, India) or MB BacT (bioMerieux India, New Delhi). BACTEC 12B vials (4 ml medium in 20 ml vial, Becton-Dickinson, Haryana, India) containing 0.1 ml PANTA were inoculated with 0.5 ml of processed specimen after cleaning the rubber septum with phenol and flushing the bottle with CO_2_. Vials were incubated at 37°C for 6 weeks, and tested for growth every 2–3 days in the first three weeks, followed by once weekly. Positive bottles were smeared when growth index reached 50. If no acid fast bacilli were seen, a gram stain was performed and blood agar inoculated to detect contamination. Contaminated vials were aspirated and treated with 4% NaOH for 15 minutes, then centrifuged at 3000g for 15 minutes, resuspended in 0.5 ml of buffer and reinoculated. Twelve vials were contaminated in the study (6%). NAP test was used for identification by diluting culture according to growth index and inoculating 1 ml into a NAP containing vial.[19] Growth in NAP was compared to growth in the original vial. NAP test was controlled using ATCC strains.

Two uninoculated 12B bottles were tested at the end of each run, at the last position, to serve as negative controls. BACTEC performance test standards (Becton-Dickenson, Haryana, India) were used twice per month and needles were removed and sterilized by autoclave weekly.

### Solid Culture

Five drops of decontaminated sputum was inoculated onto Lowenstein-Jensen slants (in-house), and incubated on a slant in the dark at 37°C for 7 weeks. Slants were examined weekly, and contaminated slants were discarded and reinoculated with decontaminated specimen. Positive growth was confirmed with ZN smear, paranitrobenzoic acid and niacin tests. Every new batch of LJ media was controlled for sterility and for growth, using *M. tuberculosis* (ATCC H37Ra) and *M. gordonae* (ATCC 14470).

### Blinding

Slides made from random number-labelled Falcon tubes, and were labelled with the same random number. A table connecting random numbers with study numbers was kept by the laboratory investigator, in a locked file.

### Data analysis

189 pulmonary and 11 extrapulmonary TB suspects were recruited as part of a larger diagnostic evaluation project. This was considered sufficient for our pilot study. Reference standard was defined as positivity on either solid or liquid culture. Data were entered by two separate operators, and manually rechecked after entry. Performance was reported as sensitivity and specificity (with 95% confidence intervals) of optimized smear as compared to LJ culture, as well as incremental yield of optimized smear as compared to direct ZN smear. Comparisons were made using Chi-Square. Analysis was performed using SPSS 15.0 software (LEAD technologies, USA).

## Results

**Figure 1:**
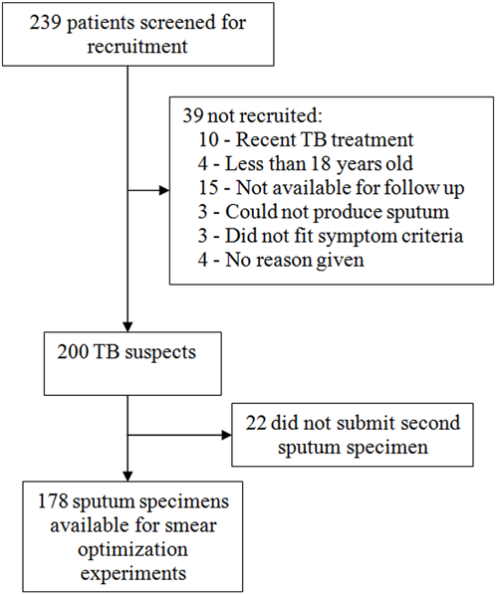
Recruitment of Study Participants.

Figure 1 shows the recruitment process and Table 2 provides a description of the study participants. 178 specimens were available from 178 pulmonary and extrapulmonary TB suspects. Patients were mostly male (109, 61.2%), outpatients (167, 93.8%) with a mean age of 40.2 years (SD 15.8) and a mean body mass index of 19.6 (SD 3.96). Sixteen (9.0%) were HIV infected and 36 (20.2%) had at least one culture positive for tuberculosis.

**Table 2:** Demographics of Study Participants.

	N	%
Total pulmonary and extrapulmonary TB suspects included in smear optimization analysis	178	100
Pulmonary TB suspects	175	98.3
Male	109	61.2
Mean Age	40.2 years	SD 15.8
Mean body mass index (N = 170)	19.6	SD 3.96
Outpatient	167	93.8
Living in Tamil Nadu state	166	93.3
Monthly family income <US$ 115	163	91.6
Experiencing cough	175	98.3
Experiencing chest pain	98	55.1
Experiencing fever	154	86.5
HIV infected	16	9.0
Either solid or liquid culture positive	36	20.2
Given treatment for TB after recruitment (N = 131)	35	26.7

### Incremental Yield of Pretreated Smears

Among 36 culture positive sputums, direct ZN stain detected 22, providing a sensitivity of 61.1% (Table 3). The direct auramine smear detected 26 (sensitivity 72.2%) for an incremental yield above direct ZN of four positives (11.1%). Both pretreated auramine smears detected the same number of positives (24/36, 66.6%), providing two additional positives (5.5% incremental yield) over direct ZN smear, but no incremental yield over direct auramine smear. There was no significant difference observed between yield among smear techniques.

**Table 3:** Incremental Yield of Smear Optimization.

	N	%	Incremental Yield Over Direct ZN Smear (%)	p value Compared to Direct ZN Smear
Total Culture Positives	36	100		
Direct ZN Smear Positives	22	61.1		
Direct Auramine Smear Positives	26	72.2	11.1	p = 0.171
Bleach Centrifuge Auramine Smear Positives	24	66.6	5.5	p = 0.494
USP Centrifuge Auramine Smear Positives	24	66.6	5.5	p = 0.494

ZN = Ziehl-Neelsen

USP = universal sediment processing

### Diagnostic Accuracy of Pretreated Smears

Direct ZN smears had 61.1% [43.5–76.4] sensitivity and 93.0% [87.1–96.4] specificity when compared to culture (Table 4). Direct auramine smears had 72.2% [54.6–85.2] sensitivity and 97.2% [92.5–99.1] specificity. There was a trend toward increased sensitivity of auramine smear, but specificity was similar, and very high. Pretreated smears were not significantly more sensitive than direct ZN or auramine smears (66.6%, [48.9–81.0]). Pretreatment actually slightly increased the number of false positives, reducing the specificity of pretreated smears (92.6% [87.1–96.4]) as compared to direct aurmine smears.

**Table 4:** Diagnostic Accuracy of Direct and Pretreated Smears as compared to Combined Solid and Liquid Culture of Sputum (N = 178).

	Sensitivity (%)	95% C.I.	Specificity (%)	95% C.I.	Positive Predictive Value (%)	95% C.I.	Negative Predictive Value (%)	95% C.I.
Direct ZN smear	61.1	43.5–76.4	93.0	87.1–96.4	68.8	49.9–83.3	90.4	84.1–94.5
Direct auramine smear	72.2	54.6–85.2	97.2	92.5–99.1	86.7	68.4–95.6	93.2	87.6–96.5
Bleach Centrifuge auramine smear (N = 174)	66.6	48.9–81.0	92.3	86.2–95.9	68.6	50.6–82.6	91.6	85.5–95.4
USP Centrifuge auramine smear	66.6	48.9–81.0	92.6	87.1–96.4	70.6	52.3–84.3	91.7	85.6–95.4

ZN = Ziehl-Neelsen

USP = universal sediment processing

CI = confidence interval

Among 16 HIV infected patients, two were culture positive. All direct and pretreated smear methods detected one out of two. There was no significant difference between methods among this small subgroup.

### Difference between chemical processing methods

There was no observed difference between bleach and USP methods (Table 4).

## Discussion

Our preliminary study evaluated two short-duration sputum pretreatment methods among pulmonary TB suspects in a large referral centre in India. We observed no additional diagnostic benefit of short duration pretreatment procedures on direct sputum smears performed using auramine staining, which is the routine method in our center. As compared to direct ZN staining, auramine is more sensitive and specific, and pretreatment does not add to this difference. Further studies of smear optimization strategies are ongoing in several countries, supported by TDR/WHO.[20]

Our study demonstrates the excellent performance of direct smears in our setting. We had a single experienced and dedicated research technician who was able to spend adequate time reviewing each slide. This may limit the generalizability of our results in national TB control program conditions, in which pretreatment may have more of an impact. Most settings in which smear optimization may be used will not have access to fluorescent microscopy, a method that is known to be more sensitive than conventional microscopy.[21]

Previous reports using short processing times have demonstrated variable, if any, benefit on smear sensitivity,[7] and as such the potential benefit of sputum pretreatment may not be realized when brief incubation procedures are used. Further investigation should use longer duration procedures.

Previous reports of benefit gained by sputum pretreatment have not contained rigorous blinding procedures, and may have overestimated the benefit of short duration pretreatments.[22] Our report included a procedure in which the laboratory technician was unaware of the previous or concurrent smear results.

HIV infected patients with TB tend to remain smear negative, so the performance of smear optimization in this group is of critical importance. Our number of HIV infected patients was too small to make valid conclusions. This again is an area for further research.

Other strengths of our study include consecutive enrollment of all TB suspects and a clear description of enrolled patients. These methods reduce the risk of selection bias and allow the reader to compare our population with TB suspects in other settings. It was an important feature of our design that specimens were split in the laboratory before comparison, such that comparisons between staining techniques (incremental yield) were made on the same sputum specimen, controlling for between sample variation. The sputum sample used for pretreatment was different from the sputum sample used for culture. Ideally, all tests would be run from the same sputum sample, but this may not be feasible due to total specimen volume.

Potential limitations of our design include the small volume of sputum (0.5 ml) used for individual smear optimization procedures. This volume is much lower than that expected in an individual diagnostic specimen, but was chosen to allow within specimen comparison between tests. It is also recognized that bleach may spontaneously deteriorate under storage, causing variation in final free chlorine concentration at the time of use.[23] Our study did not control for this factor.

Our procedure could be improved further by three means. Homogenization of sputum by prolonged vortexing with glass beads, although associated with increased risk to technicians, may have distributed bacilli more evenly and may have increased yield.[24] The volume of sputum used for each test was small and could be increased by performing fewer tests on each specimen. It is well recognized that larger volume sputum specimens are associated with increased smear positivity.[25] There is new evidence that conventional centrifugation techniques may not be adequate to separate mycobacteria from sputum, and that longer centrifugation time may be required.[26]

A recent report from Kenya, with a similar design to ours, suggested that USP pretreatment was not superior to NALC as determined by culture yield.[27] Direct smear was not compared. Centrifugation with bleach pretreatment significantly increased smear yield in Ethiopia, although this population had a higher (background) HIV prevalence than ours.[28]

One important challenge in the use of sputum pretreatment with bleach is the loss of viability required for mycobacterial culture. Because most patients in TB endemic countries do not have access to quality controlled culture facilities, inactivation is not yet a significant operational challenge. Until culture facilities become more widely available, this should not restrict the application of inactivating pretreatment strategies which may improve yield.

In summary, our pilot data suggest that short duration sputum pretreatments provide no additional diagnostic benefit in India. Further work, using larger sample sizes, is needed to confirm this, and also to evaluate long duration pretreatment approaches.
